# Timely, Dignified, Efficient: Modernizing Verification of Death in Switzerland

**DOI:** 10.3389/ijph.2026.1609069

**Published:** 2026-04-14

**Authors:** Sarah Maria Esther Jerjen

**Affiliations:** Faculty of Health Sciences and Medicine, Universitat Luzern, Lucerne, Switzerland

**Keywords:** emergency medical services, health system efficiency, Switzerland, task shifting, verification of death

**The IJPH series “Young Researcher Editorial” is a training project of the Swiss School of Public Health**

In many health systems, only physicians are legally allowed to verify a death, which remains a defining feature of emergency care organization. This applies even in countries with highly developed emergency medical services. Switzerland is a prime example: under national medico-legal rules, verification is reserved to physicians even when a paramedic is already on scene [1]. In most Swiss cantons, not even an on-call emergency doctor is authorized to perform this task; instead, they must call another physician, often a general practitioner or official doctor [2] while families and first responders wait on scene. On call physicians must then spend precious time on a routine procedure that could be safely managed by others [2–5].

This established institutional approach has increasingly diverged from international practice because it is inefficient and may appear undignified to the deceased person’s family and observers [5]. In Switzerland, nearly a third of persons who have had out-of-hospital cardiac arrests exhibit obvious signs of death [6] and emergency services often recognize the futility of resuscitation. In these cases, waiting for another physician is unnecessary and prevents emergency personnel from responding to new calls [5]. Families waiting for verification must endure longer periods of uncertainty and distress [7]. Framing verification as a public health issue highlights that a medico-legal formality can influence system performance and service delivery.

The WHO promotes structured redistribution of responsibilities within health teams, which they call “task shifting”—a process that delegates routine, protocol-driven duties to trained healthcare workers, while reserving complex decisions for physicians. Task shifting improves efficiency, expands coverage, and preserves quality of service [8]. Verification of death is a textbook candidate for task shifting: it is a standardized, procedural act that can be safely managed by trained nurses or paramedics operating under clear criteria, leaving physicians to later certify the *cause* of death.

International models demonstrate how this can work in practice. Many health systems distinguish between verification of death (VoD) and medical certification of cause of death (MCCD) [3]. Verification is based on standardized clinical signs including rigor mortis, lividity, or asystole [4], while certification requires diagnostic judgment and appropriately remains a physician’s responsibility [3]. In the UK, Ireland, Australia, France, South Africa, Canada, and several US states, paramedics or nurses are authorized to perform VoD when clear criteria are met. Later, physicians complete the MCCD, which is not time-critical and can be done during routine practice hours [3–5, 7]. Evaluations from these jurisdictions consistently show no clinically meaningful differences in accuracy between VoD made by physicians and trained non-physicians, if the appropriate protocols are followed [9]. These examples show that modernizing VoD speeds the process, preserves the dignity of the deceased and their family, and protects scarce medical resources without sacrificing safety [3].

In Switzerland, the costs of restrictive rules are clear. Emergency physicians, paramedics, and nurses regularly encounter death in the course of resuscitations, trauma, and end-of-life care, yet are legally excluded from performing VoD [6]. Instead, the task usually falls to doctors who may perform it rarely, often under difficult circumstances such as during night callouts [2]. The consequences of this arrangement fall unequally; for example, families in rural regions often face far longer waits for verification than those in urban centers, compounding healthcare inequities at life’s end [2, 6].

Historically, this physician-only model evolved at a time in which non-physician roles were less formalized. This burdens general practice while sidelining professionals with far greater exposure to secure signs of death. Given the conditions under which healthcare workers are trained and governed today, we no longer need to rely on professional exclusivity to ensure high quality care. In practice, the competence of health professionals is secured by clear criteria, training, and repeated experience—all of which emergency healthcare workers already possess or can acquire through structured training [9].

Expanding the power to verify death to appropriate professionals will require us to surmount a series of barriers, including medico-legal liability concerns, tensions between the boundaries of medical professions, and reforming legislation to separate VoD from MCCD. The task requires we create and administer a nationally accepted framework for training, certification, and quality assurance.

Switzerland should, however, align with international best practice and WHO guidance by reforming legislation to separate VoD from MCCD. National protocols should define the clear signs of death, exclusions, and escalation procedures. The professionals we authorize to perform VoD—paramedics, nurses, and emergency physicians (in all regions)—should be trained, certified, and subject to regular audit [3]. Physicians will continue to perform MCCD, focusing their expertise where it is most valuable. Safeguards could include digital documentation, telemedicine support, and structured communication scripts for use with bereaved families [3].

Precedents for task shifting already exist in Switzerland, which has expanded roles in other domains. For example, midwives can prescribe medications, advanced practice nurses manage chronic conditions, and pharmacists administer vaccines. Task shifting for death verification is a natural next step.

Authorizing trained non-physicians to perform VoD under standardized criteria will improve timeliness, reduce distress, and free scarce medical resources. Switzerland should begin pilot reforms in selected regions, evaluate outcomes including safety, timeliness, and family experience, and scale up successful models so that cantons can adopt them. Modernizing VoD is not about diminishing the physician’s role; it is about adapting institutional frameworks to support responsive, equitable, and sustainable health systems.
